# Trends of Prevalence of Uncontrolled Risk Factors for Cerebrocardiovascular Disease: Southern Italy from 1988/9 to 2008/9

**DOI:** 10.1155/2016/6087981

**Published:** 2016-04-24

**Authors:** Vincenzo Capuano, Norman Lamaida, Ernesto Capuano, Rocco Capuano, Eduardo Capuano, Gianfranco Mazzotta

**Affiliations:** ^1^Cardiology Department, “G. Fucito” Hospital, Azienda Ospedaliera Universitaria di Salerno, 84085 Mercato San Severino, Italy; ^2^Cardiology Department, “S. Andrea” Hospital, 19124 La Spezia, Italy

## Abstract

The aim of this study was to determine the trends of cardiovascular risk factor prevalence between 1988/9 and 2008/9 in the 25–74-year-old population in an area of Southern Italy.* We compared* three cross-sectional studies conducted in random population samples, in 1988/9, 1998/9, and 2008/9 in Salerno, Italy. The methodology of data collection (lipid profile, systolic and diastolic blood pressure, glycaemia, and smoking) and conducting tests which the population underwent during the three phases was standardized and comparable. Prevalence of diabetes, hypertension, hypercholesterolemia, and smoking was calculated and standardized for age. A total of 3491 subjects were included. From 1988/9 to 2008/9, in males, the prevalence of all four risk factors was reduced. In women, there was a clear reduction of hypertension, a similar prevalence of hypercholesterolemia, and an increase of smoking and diabetes. In the area of Salerno, our data confirm that the global prevalence of the major risk factors is decreasing in men, but their absolute values are still far from optimization. In women, diabetes and smoking showed a negative trend, therefore requiring targeted interventions. These data are now used as a base for executive targeted programs to improve prevention of cardiovascular disease in our community.

## 1. Introduction

In both males and females, cardiovascular mortality and morbidity have been progressively decreasing since the 1970s, in Italy as well as in other western countries [1, 2]. Such reduction is the result of several factors and actions but is especially due to a better control of risk factors [3]. Data are needed to control and improve the trend of prevalence of cardiovascular risk conditions.

In Italy, the prevalence of risk factors has been documented since the early years following World War II. The “Seven Country Study” [4] was conducted in Calabria, and the “Study of the Nine Communities” (Italian multicenter study) involved nine areas in 9 regions [5, 6]. Afterwards, data were provided by the MONICA Project [7], covering two geographical areas, and, more recently, from the Osservatorio Epidemiologico Cardiovascolare (OEC) [8]. Studies of smaller areas provided patchy data, but prospective local studies still are essential to plan targeted interventions for primary prevention. Studying cardiovascular risk factors prospectively implies the use of common methodologies and standardized measurements, so that data may be comparable at different times.

The “Valle dell'Irno Prevention” (VIP) project is collecting epidemiological data every year since the end of the 1980s, by the same team (same coordinator) and using the same methodologies, aimed at comparing general population samples (25–74 years). These data provide the possibility of creating twenty-year trends of many risk factors. Hereby, we are presenting the trends in our area as for hypercholesterolemia, hypertension, diabetes, and cigarette smoking.

## 2. Methods

We compared the results of three epidemiological surveys performed in Southern Italy, in a Mediterranean region (Campania), and particularly in two areas near the city of Salerno. We compared the data collected as part of “Montecorvino Rovella Project” [9] (PMR), 1988-89, and cross-sectional data from the two phases of the “Valle dell'Irno Prevention (VIP) project”: 1988-89 (first phase) [10] and 2008-09 (second phase). In these three investigations, a sample taken from people between 25 and 74 years old was studied and subdivided, by sex, into 5 decades of age: 25–34, 35–44, 45–54, 55–64, and 65–74 years. All these citizens were randomly enlisted from the electoral rolls and underwent blood tests after overnight fasting.

The methodology of data collection during the three phases is standardized and comparable and already described [9–11]. PMR and VIP projects were conducted by the same working group and by the same coordinator. Both geographical areas are similar and both are within 20 km from Salerno. The socioeconomic conditions of the rural populations and their recent industrial development are similar. The proportions between urban and rural population were similar.

### 2.1. PMR Project Design

Aiming to analyze the prevalence of cardiovascular risk factors in an area of the Campania region at the end of the 1980s, this study was conducted between 1988 and 1989, after inviting a randomized statistical sample representative of the area. Randomized samples included 1500 subjects, 300 (150 males and 150 females) for each decade. Only 1091 subjects (569 females and 522 males) were examined with a total participation of 72.7% (75.9% for females and 69.6% for males).

### 2.2. VIP Project Design

This is a program of cardiovascular prevention in a population of Valle dell'Irno, controlled by Mercato San Severino Hospital, aiming to know the clinical data and biohumoral parameters of the resident population, as well as defining the tendency over time of the main cardiovascular risk factors in the area near Salerno. The VIP study has collected epidemiological data on cardiovascular risk factors in two phases: 1998/99 and 2008/09. It is part of the CINDI Program, a WHO study [12, 13], and has contributed to the Global Burden of Metabolic Risk Factors of Chronic Diseases Collaborating Group study [14].

Both surveys include 1200 subjects, 600 males and 600 females, age ranging from 25 to 74 years (120 subjects per decade), randomized from the electoral rolls of the towns of Mercato San Severino and Baronissi, near Salerno, in Southern Italy. In a randomized way, we compiled three lists, each one of 120 subjects, and then subdivided them into decades of sex and age. The recruitment from the first list was realized by letter of invitation; in the case of impossibility or refusal, the subject was replaced by another of the same age and gender from the second list and again in case of failure by someone from the third list. This type of procedure of recruitment was suggested by the manual of the rules of MONICA Project: MONICA Cardiovascular Diseases [15].

During all phases, the subjects underwent

- physical examination,
- recording of blood pressure,
- anthropometric measurements (weight, height, and waist-hip ratio),
- electrocardiogram,
- laboratory tests (total cholesterol, LDL cholesterol, HDL cholesterol, triglycerides, blood glucose, hemochrome, plasma insulin, fibrinogen, creatinine, and C3).

Fasting venous blood was obtained in the seated position without stasis, after overnight fast. Quality control of the laboratory was Bio-Rad (in 1988/89 and 1998/99) and VEQ (University Hospital of Bologna, Policlinico S. Orsola-Malpighi) in 2008/09. Clinical history was taken by a physician, focusing on cardiovascular disease. Cigarette smoking, physical activity, occupation, level of education of both the subject and the partner, civil status, and regular use of pharmacological therapy were investigated by questionnaire. Blood pressure was measured following the criteria of the World Health Organization [16]. The pressure values, in all three of the detections, were detected using a mercury sphygmomanometer (F. Bosch). We measured the blood pressure twice; the first value was obtained with the patient sitting after at least 5-minute waiting and relaxing. We considered the mean of two determinations. Total cholesterol was determined in all three surveys, using an enzymatic method. Glycaemia was always determined by hexokinase method, in both cases with model Olympus AU640.

## 3. Definitions

### 3.1. Uncontrolled High Blood Pressure

The measured systolic blood pressure was ≥140 mmHg and the diastolic blood pressure was ≥90 mmHg, based on an average of up to two measurements. Persons defined as having uncontrolled high blood pressure may or may not have been taking medications.

### 3.2. Uncontrolled High Cholesterolemia

Values of cholesterolemia ≥240 mg/dL. Persons defined as having uncontrolled high cholesterolemia may or may not have been taking medications.

### 3.3. Smoker

Smoking habit was determined by an interview. We have data on the number of cigarettes smoked and for ex-smokers on how long they had stopped smoking and how many cigarettes they smoked. We have data on passive smoking, in the family and in the workplace. In the survey of 2008, the concentration of pulmonary CO was also evaluated. All subjects who smoked at least one cigarette every day were considered smokers.

### 3.4. Uncontrolled High Glycaemia

Fasting values of glycaemia ≥126 mg/dL. Persons defined as having uncontrolled high glycaemia may or may not have been taking medications.

## 4. Statistical Analysis

Data are expressed as mean ±1 standard deviation. As far as risk factors are concerned, the prevalence is given, in terms of percentage. Data have been standardized using the direct method considering the European population standards of reference (2008).

To compare the means among the three groups, we used one-way analysis of variance and Bonferroni's test for the differences among the groups. Chi-square analysis was used to compare prevalences.

## 5. Results

**Figure 1:**
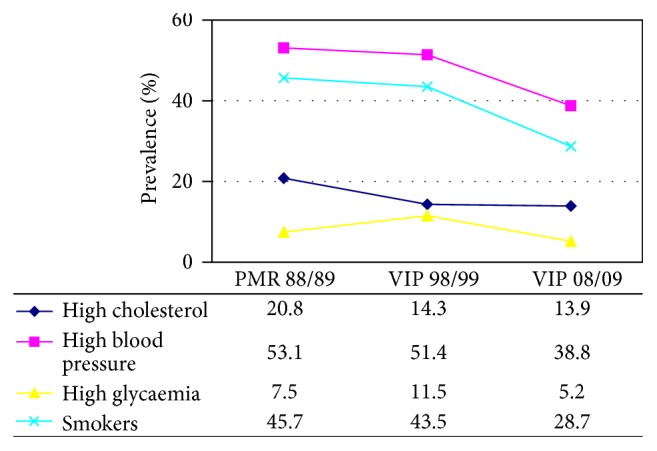
Prevalence of uncontrolled major risk factors from 1988/9 to 2008/9. Standardized data for the European population: males.

**Figure 2:**
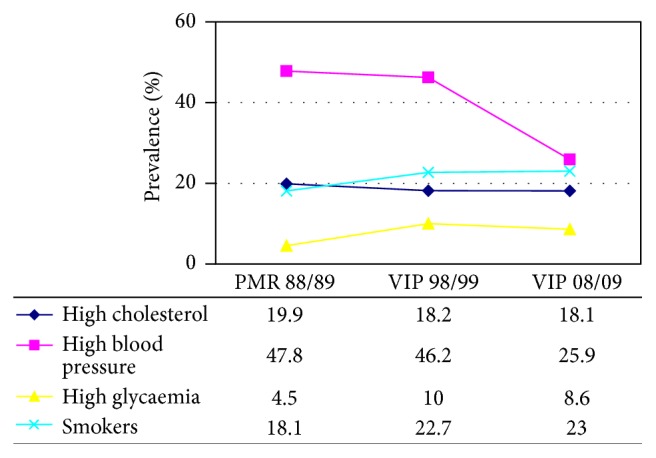
Prevalence of uncontrolled major risk factors from 1988/9 to 2008/9. Standardized data for the European population: females.

Table 1 (males) and Table 2 (females) reported the prevalence of major risk factors in the three periods examined as well as the percentage of medically treated persons and of effective treatment and the percentage of subjects at target.  Figure 1 (males) and Figure 2 (females) show the trend of the prevalence of uncontrolled risk factors: high blood pressure, high cholesterolemia, smoking, and high blood glucose level. In the period 1998/99–2008/09, in the male population, the prevalence of individual risk factors was similar. The risk factor with the highest prevalence was and still remains high blood pressure and that with the lowest prevalence was and still remains high glycaemia.

In women, the prevalence of risk factors in the last survey (2008/09) has the same sequence of the males, while at the end of the 1980s smokers were fewer than women with high cholesterolemia. Nowadays, in both genders, smokers have a higher prevalence than hypercholesterolemics. Furthermore, while in males the prevalence of all four risk factors was reducing with time (in all cohorts, with statistically significant differences), in women there was a clear reduction of the sole hypertension (statistically significant difference). In women, the prevalence of high cholesterolemia remained essentially unchanged, while we recorded an increase through years of smokers and women with high glycaemia (statistically significant differences).

Table 3 (males) and Table 4 (females) show the comparison of the mean values of the variables under consideration reported in the three surveys. For cholesterolemia, whereas in males there is a slight but progressive reduction of the mean values (statistically significant), in females the values remain unchanged. For fasting blood glucose, there is a clear increase of the mean values in both genders in the period 1988/89 to 1998/99, with a subsequent reduction in the next decade that does not reach the values recorded in 1988/89.

## 6. Discussion

In this study, we have monitored the prevalence of cardiovascular risk factors in the Campania region through 20 years, in the period 1988/89–2008/09. In our opinion, major observations are as follows:

- There has been a global reduction of cardiovascular risk burden, and this is aligned with other reports, in Italy and in other countries of the western world [1, 3, 4, 17]. Our data, however, show a clear positive trend for all four risk factors examined only in males, whereas in females there has been a reduction in the prevalence of hypertension. The prevalence of hypercholesterolemia remains essentially unchanged, and there has been an increase of prevalence of hyperglycaemia and smoking in women. Cigarette smoking becomes the second risk factor in order of prevalence, with values similar to the prevalence of hypertension. This disappointing increase in the prevalence of smokers among women may reflect the underestimation of the risk of ischemic heart disease in females: attention is often posed only in advanced ages, or treatment is less aggressive than in males [18–20].
- The mean blood pressure of the population, especially during the last decade, has decreased, as well as the prevalence of subjects with high blood pressure; therefore, in both genders, the number of hypertensive patients at target has increased. This finding is probably the result of several factors: the special attention given to this risk factor, due to lifestyle change or lifestyle modification, and possibly also the recent availability of new drugs, which are more effective and with fewer side effects. Despite this, however, still 38.8% of men and 25.9% of women are still hypertensive. In the examined population, the mean systolic blood pressure is approximately 2 mmHg higher than the world mean (SBP worldwide in 2008 was 128.1 mmHg in men and 124.4 mmHg in women [20]). Epidemiological studies have shown that from 1988 to 2008 a reduction of the mean arterial pressure is evident throughout the world, but in smaller proportion in Western Europe [21]. Similarly, in our area, mean systolic and diastolic blood pressure have fallen in twenty years, by about 2 mmHg in men, and in women this reduction has only been observed for diastolic BP. This result, however, is of utmost importance when you consider that even a reduction of only 2 mmHg in the global population produces a decrease in stroke mortality of 6% and in ischemic heart disease of 4% [22]. This is even more important in Campania, because women living there have always shown a particularly high incidence of stroke [23]. Furthermore, a higher level of baseline risk is not due to treatment per se, the risk being similar in uncontrolled and untreated hypertension. Adjustment for risk factors reduces the risk only in controlled hypertension, suggesting that there may be structural alterations scarcely reversible by antihypertensive treatment [24].
- The trend of cholesterolemia is favorable, similar to that registered in other parts of the world in the same period [14]. We suppose that the decrease in uncontrolled hypercholesterolemia was mainly due to medication such as statins. We have observed a more favorable trend in males, and this result might be once again justified by a greater attention for the male gender: there is, in fact, a difference between the two genders in the percentage of treated hypercholesterolemics (48% in men and 39.1% in women) and in reaching an acceptable target (91% in men and 80.2% in women). A recent meta-analysis has shown that adherence to preventive treatment is poorly and little related to the class of drug, suggesting that side effects are not the main causes of undertreatment. General measures, rather than class-specific ones, are needed to improve adherence [25].
- As for the smoking habit, we have registered a clear reduction of smokers and heavy smokers in males and a slight, steady increase of female smokers. The number of cigarettes smoked daily increased in female smokers and decreased in male smokers. This confirms what is happening all over Italy for men: a continuous decrease in male smokers since the 1960s. This is in contrast with the national trend of women: starting from the 1960s until the late 1980s, there has been an increase of female smokers, and only from the early 1990s a slightly decreasing trend started [26]. This data shows the delay in changing trends, which has often been recorded in southern areas in comparison with the regions of the north. It will be essential to plan executive programs aimed at decreasing smoking among women so that we can reverse the trend also in our area.
- The trends of the prevalence of uncontrolled glycaemia in both genders show an increase in the transition from 1988/89 to 1998/99, with a subsequent reduction in the next decade. It is likely that between the second and third decades of life eating habits have improved, and also there have been new drugs available.

Globally, over the last twenty years, there has been a slight decrease in men and an increase in women. On the other hand, the worldwide data show a slight increase in both genders, with an annual increase of 0.03 mmol/L for women and 0.07 mmol/L for men [27]. The hope is that the slight reduction in hyperglycaemic males may be the first sign of a change and that this trend can be consolidated over time and extended in the females as well.

The data confirm that the global prevalence of the major risk factors is steadily decreasing in men, but their absolute values are still far from optimal. This unsatisfactory situation may explain why Campania is the Italian region with the largest number of preventable deaths for both genders: 62.5% for males (48.5% in Italy) and 21.2% for females (13.8% in Italy) [28]. In particular, very little has been done for the reduction of the prevalence of risk factors in women, and there is a need for specific executive programs targeted at the female gender.

The high incidence of morbidity and mortality related to cardiovascular disease is a major problem in all western countries, but the risk is even greater in the Campania region. Thus, collecting epidemiological data prospectively is essential, and such work is meaningful if the information will serve as a database to build and maintain cardiovascular prevention programs targeted to the area. In this sense, it is increasingly necessary to raise awareness among the clinical cardiologists, to consider the epidemiological data as the essential tools to implement their own strategies of intervention.

## Figures and Tables

**Table 1:** Prevalence of uncontrolled major risk factors from 1988/9 to 2008/9. Standardized data for the European population: males.

Risk conditions	Group 1 1988-89 (*n* = 522)	Group 2 1998-99 (*n* = 600)	Group 3 2008-09 (*n* = 600)	*p*	1 versus 3	1 versus 2	2 versus 3
Hypercholesterolemia							
Uncontrolled high cholesterol	20.8%	14.3%	13.9%	0.003	<0.05	<0.05	NS
Hypercholesterolemic	20.8%	16.4%	21.1%	NS			
Hypercholesterolemic treated	—	2.5%	40.8%				
Treated at target	—	84%	91%
Hypercholesterolemic at target	—	12.8%	37%
Hyperglycaemia							
Uncontrolled high glycaemia	7.5%	11.5%	5.2%	0.000	NS	<0.05	<0.05
Diabetics	9.1%	11.9%	7%	0.016	NS	NS	<0.05
Treated diabetics	30.1%	30.2%	65.7%				
Treated at target	13%	11.1%	39.1%
Diabetics target	5%	3.7%	25.7%
Smokers							
Current smokers	45.7%	43.5%	28.7%	0.000	<0.05	NS	<0.05
Ex-smokers	33.1%	27.7%	32%	NS			
Nonsmokers	21.2%	28.8%	39.2%	0.000	<0.05	<0.05	<0.05
Hypertension							
Hypertensives	55.8%	54.2%	45.2%	0.000	<0.05	NS	<0.05
Uncontrolled high blood pressure	53.1%	51.4%	38.8%	0.000	<0.05	NS	<0.05
Treated hypertensives	30%	30.6%	26.6%				
Effectively treated	16.1%	16.9%	53.3%
Hypertensive target	5%	5.2%	14.2%

Uncontrolled high cholesterol: patients with cholesterol ≥240 mg/dL, treated and not treated.

Hypercholesterolemic subjects: uncontrolled high cholesterol + subjects on cholesterol-lowering therapy.

Hypercholesterolemic subjects treated: hypercholesterolemic treated/hypercholesterolemic subjects.

Patients at the target: treated target (cholesterol < 240 mg/dL)/patients treated.

Hypercholesterolemic at the target: hypercholesterolemic at the target/hypercholesterolemic.

Uncontrolled high glycaemia: glycaemia ≥ 126 mg/dL, treated and not treated.

Diabetic patients: uncontrolled high glycaemia + patients treated with hypoglycaemic drugs.

Diabetic treated: treated/diabetic subjects.

Diabetic treated to target: treated target (glycaemia < 126 mg/dL)/diabetics treated.

Diabetic at target: diabetic target/diabetics.

Uncontrolled high blood pressure: SBP ≥ 140 and/or DBP ≥ 90 mmHg, treated and not treated.

Hypertensive patients: uncontrolled high blood pressure + subjects in antihypertensive therapy.

Hypertensive therapy effective: subjects treated with target values/subjects treated.

Effectively treated: treated target/subjects treated.

Hypertensive at target: treated hypertensives with target values/hypertensives.

**Table 2:** Prevalence of uncontrolled major risk factors from 1988/9 to 2008/9. Standardized data for the European population: women.

Risk conditions	Group 1 1988-89 (*n* = 569)	Group 2 1998-99 (*n* = 600)	Group 3 2008-09 (*n* = 600)	*p*	1 versus 3	1 versus 2	2 versus 3
Hypercholesterolemia							
Uncontrolled high cholesterol	19.9%	18.2%	18.1%	NS			
Hypercholesterolemic	19.9%	18.9%	25.8%	0.000	<0.05	NS	<0.05
Hypercholesterolemic treated	—	7.9%	39.1%				
Treated at target	—	46.7%	80.2%
Hypercholesterolemic at target	—	3.7%	37%
Hyperglycaemia							
Uncontrolled high glycaemia	4.5%	10%	8.6%	0.002	<0.05	<0.05	NS
Diabetics	8%	10.3%	9.3%	NS			
Treated diabetics	35.2%	36%	61.3%				
Treated at target	8.3%	8.1%	12.3%
Diabetics at target	3%	2.9%	7.5%
Smokers							
Current smokers	18.1%	22.7%	23%	NS			
Ex-smokers	6.9%	8.3%	10.6%	NS			
Nonsmokers	75%	69%	66.4%	0.005	<0.05	<0.05	NS
Hypertension							
Hypertensives	51%	48.5%	34.1%	0.000	<0.05	NS	<0.05
Uncontrolled high blood pressure	49.1%	46.2%	25.9%	0.000	<0.05	NS	<0.05
Treated hypertensives	6.8%	37.5%	39.3%				
Effectively treated	13%	12.6%	61.2%
Hypertensive at target	5.2%	4.7%	24%

Uncontrolled high cholesterol: patients with cholesterol ≥ 240 mg/dL, treated and not treated.

Hypercholesterolemic subjects: uncontrolled high cholesterol + subjects on cholesterol-lowering therapy.

Hypercholesterolemic subjects treated: hypercholesterolemic treated/hypercholesterolemic subjects.

Patients at the target: treated target (cholesterol < 240 mg/dL)/patients treated.

Hypercholesterolemic at the target: hypercholesterolemic at the target/hypercholesterolemic.

Uncontrolled high glycaemia: glycaemia ≥ 126 mg/dL, treated and not treated.

Diabetic patients: uncontrolled high glycaemia + patients treated with hypoglycaemic drugs.

Diabetic treated: treated/diabetic subjects.

Diabetic treated to target: treated target (glycaemia < 126 mg/dL)/diabetics treated.

Diabetic at target: diabetic target/diabetics.

Uncontrolled high blood pressure: SBP ≥ 140 and/or DBP ≥ 90 mmHg, treated and not treated.

Hypertensive patients: uncontrolled high blood pressure + subjects in antihypertensive therapy.

Hypertensive therapy effective: subjects treated with target values/subjects treated.

Effectively treated: treated target/subjects treated.

Hypertensive at target: treated hypertensives with target values/hypertensives.

**Table 3:** Comparison between the mean values ± 1 standard deviation of the main cardiovascular risk factors. Data collected in 1988/89–2008/09: males, age 25–74 years. Data standardized to the European population.

Risk factors	Group 1 1988-89 (*n* = 522)	Group 2 1998-99 (*n* = 600)	Group 3 2008-09 (*n* = 600)	*p*	1 versus 3	1 versus 2	2 versus 3
Systolic blood pressure (mmHg)	130.2 ± 15.3	132.7 ± 16.8	130.9 ± 16	0.025	NS	<0.05	NS
Diastolic blood pressure (mmHg)	80.5 ± 9.5	84.1 ± 9.9	78.8 ± 9	0.000	<0.05	<0.05	<0.05
Total cholesterol (mg/dL)	205.2 ± 47.1	200 ± 39.3	197.9 ± 40.2	0.009	<0.05	NS	NS
Glycaemia (mg/dL)	93.7 ± 30.1	105.6 ± 24.4	96.8 ± 25.8	0.000	NS	<0.05	<0.05
Cigarettes smoked N. (in smokers)	22.1 ± 11.9	19.2 ± 11.1	16.6 ± 11.5	0.000	<0.05	<0.05	<0.05

**Table 4:** Comparison of the mean values ± 1 standard deviation of the main risk factors and risk conditions, collected in 1988/89 and 2008/09: females, age 25–74 years.

Risk factor	Group 1 1988-89 (*n* = 569)	Group 2 1998-99 (*n* = 600)	Group 3 2008-09 (*n* = 600)	*p*	1 versus 3	1 versus 2	2 versus 3
Systolic blood pressure (mmHg)	128.8 ± 16.7	131.7 ± 16	126 ± 16.7	0.000	<0.05	<0.05	<0.05
Diastolic blood pressure (mmHg)	78.7 ± 11.5	83.1 ± 9.2	76.1 ± 9.1	0.000	<0.05	<0.05	<0.05
Total cholesterol (mg/dL)	203.1 ± 42.5	199 ± 37.1	203.3 ± 39.3	0.118			
Glycaemia (mg/dL)	87.4 ± 16.7	103.2 ± 26.6	98.9 ± 29.9	0.000	<0.05	<0.05	<0.05
Cigarettes smoked daily N. (in smokers)	9.1 ± 6.8	10.7 ± 7.1	12.1 ± 7.2	0.000	<0.05	<0.05	<0.05
